# Basis, Process and Outcomes of a Student Involvement Project for Curriculum Review at the Imperial College School of Medicine

**DOI:** 10.30476/JAMP.2022.94921.1613

**Published:** 2022-07-01

**Authors:** Sharan J Kapadia, Lana Al-Nusair

**Affiliations:** 1 Imperial College School of Medicine, London, UK

**Keywords:** Education, Medical, Curriculum, Students

## Abstract

**Introduction:**

To help create a Bachelor of Medicine, Bachelor of Surgery (MBBS) curriculum centred around the student voice, the Imperial College School of Medicine (ICSM) recruited two medical students for a two-week student-staff collaboration in Summer 2019 for its wider curriculum review. This write-up discusses the background, processes, and outcomes of the collaboration and includes some student reflections.

**Methods:**

The team comprised a member of the faculty and two medical students (the authors). We met daily for two weeks and focussed on the Bioregulatory Systems (BRS) module of Year 1. There were three key areas of work: learning objectives, large-group sessions, and small-group sessions. Each aspect involved planning, implementation, and reflection. For example, learning objectives were recategorized and reorganised, students fed back on a new slide template for large-group sessions, and new small-group sessions were designed. Feedback from the staff was collected verbally, and the medical students submitted feedback in the form of a mid-project interview, a post-project report, and informally.

**Results:**

We achieved such outcomes as reorganising and refining learning objectives, improving large-group teaching sessions, and refining and creating small-group teaching sessions. Following the collaboration, we had a debrief session.

**Conclusions:**

This collaboration was highly valuable for both students and faculty; the feedback revealed that the ideas, discussions, and outputs had a substantial impact. Overall, student-staff collaboration will become increasingly valuable as we emerge from COVID-19; we hope this write-up informs and inspires more 'students as partners’ projects worldwide.

## Introduction

Traditionally, higher education has followed a hierarchical *‘teachers teach, students learn’* model, an ideology that persists to this day ( 1 ). In contrast, the ‘Students as Partners’ model encourages student-staff collaboration, where students benefit from improved engagement, motivation, agency, and communication skills, and the faculty benefit from rich, real-time student feedback ( 2 ). One application of ‘Student as Partners’ is curriculum review – ‘student-centeredness’ is the first of Harden’s SPICES criteria (Student-centred, Problem-based, Integrated, Community-based, Electives, Systematic) for progressive curricula ( 3 ).

The Curriculum Review at ICSM involved an extensive restructure of the MBBS course over several years. In summer 2019, ICSM organised this Student Involvement Project to make progress on the review by involving medical students. The core team comprised a member of faculty (Dr James Moss) and two medical students (authors), who were selected following a competitive application process. This write-up aims to discuss the key processes and outcomes of the project, as well as some reflections. It hopes to inspire and inform the implementation of similar ‘Students as Partners’ projects worldwide.

As we emerge from the COVID-19 situation, collaborative work remains the key ( 4 ). Partnerships between medical students and faculty will be invaluable; these have the potential to produce efficient and creative results based on diverse perspectives and provide medical students an opportunity to share responsibility for the work.

## Methods & Results

### Overview

As medical students, we were given introductory information during an initial scoping meeting. Following this, our team met on campus, daily, for two consecutive weeks. The first task was restructuring and reorganising learning objectives for ‘Bioregulatory Systems’ (BRS), a module within the year-one MBBS curriculum. Secondly, we worked on improving large group teaching sessions with a focus on lecture slide design. Lastly, we refined and designed small-group teaching sessions. Feedback from the staff was collected verbally, and students submitted feedback in a mid-project interview, a post-project report, and informally.

### Task 1 – Learning objectives

Learning objectives are specific, measurable statements of observable learner behaviour or actions ( 5 ). At ICSM, learning objectives are student-facing via the online platform *Sofia*. There are Intended Learning Objectives (ILOs) at various levels: MILOs (module-level), TILOs (topic-level), and SILOs (session-level). Our focus was on TILOs.

**Figure 1:**
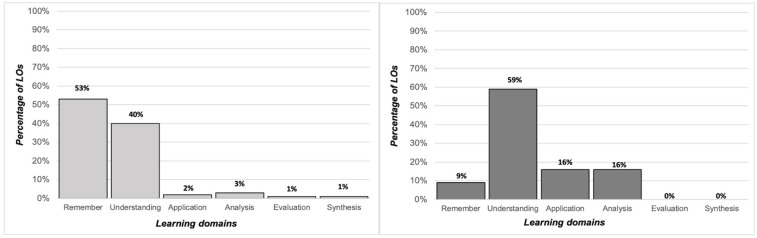
Comparison of TILO distribution between previous curriculum (left) and new curriculum (right)

The task began when the BRS topic leads (for Cardiovascular, Respiratory, Alimentary, Musculoskeletal, and so forth) submitted a reduced number of draft TILOs, 447 in total, as part of the curriculum review, to reflect the changed weightings. However, these draft TILOs were submitted in different formats and styles, not uniformly categorised, and occasionally overlapped. According to Chatterjee and Corral, learning objects should be uniform, categorised, specific, and distinct ( 5 ). Moreover, 447 was still too many TILOs for the curriculum. Our role was, therefore, to firstly reduce their number, secondly to ensure the TILOs met Chatterjee and Coral’s criteria, and thirdly to match TILOs to planned changes in assessment; exams in the new curriculum were shifting away from factual recall in favour of understanding, analysis, and application (Figure 1). This involved ‘instructional alignment’ - precise matching between instruction and assessment ( 6 ).

We reduced the number of TILOs either by combining TILOs or by reducing detail by generalising the TILOs. This required a balance between providing enough granularity without generating an excessive number of learning objectives. The result was a set of 40 TILOs. To generate consistent and uniform TILOs, we mapped them to verbs from Bloom’s taxonomy ( 7 ). This required us to consider instructional alignment (Figure 1) by avoiding too many first-order skills e.g., ‘recall’/‘define’. One example of reducing number and improving consistency was combining the three, verbless, TILOs: “organisation of nervous system (i) – structural divisions”, “organisation of nervous system (ii) – central, peripheral and autonomic” and “organisation of nervous system (iii) – CNS” into a single TILO with clear Bloom’s taxonomy verbs ( 7 ): “Compare and contrast the structure of the central, peripheral and autonomic (sympathetic and parasympathetic) nervous systems”.

Categorisation was challenging because the draft TILOs were organised in different ways. For example, gastroenterology was categorised by *Anatomical Structure & Function, Signalling & Regulation, Physiology & Homeostasis* etc., whereas neurology used *Structure and Function, Communication, Organisation,* etc. After much thought, our final categorisation was: ‘structure & function’, ‘pathology’, ‘clinical features’, and ‘clinical management’ which also minimised the overlap for specificity and distinctness. All 40 TILOs fit into one of these categories. Following a review and feedback session with the lead faculty, the 40 TILOs were approved, uploaded to *Sofia* and underpinned 1st-year BRS teaching in 2019-2020. 

### Task 2 – Improving large-group teaching sessions

**Figure 2:**
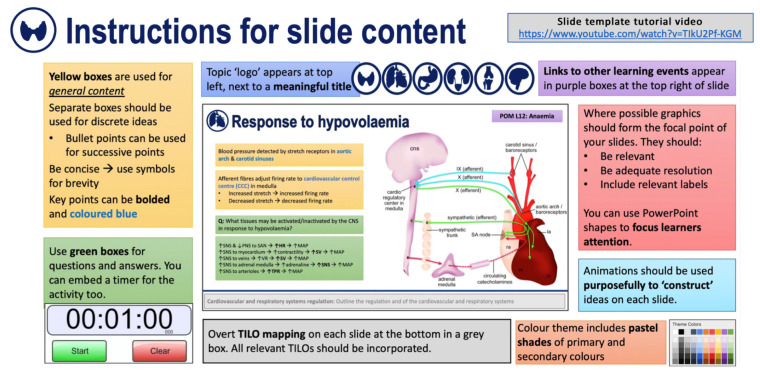
‘Instructions for slide content’ slide template (centre) surrounded by explanation of each component

Reflecting on our 1st-year lectures, we recalled the importance of consistent and effective lecture slides for student engagement. In their post-lecture feedback, students from the previous curriculum felt that effective slides “were linked to learning outcomes” and that “consistent slides made the course flow better”. Since only some modules used consistent slides, students wished that “other modules would adopt this strategy”. Dr James Moss had developed a slide template to improve the quality of the slides and maintain consistency, which we fed back on as part of the student involvement project. The template (Figure 2) is based on student feedback and multimedia design theory ( 8 , 9 ). It describes 8 key aspects of effective slides: a logo with a title, yellow boxes for general content emphasising conciseness, green boxes for questions and answers, TILO mapping in grey boxes to ensure students are aware which TILO is being taught, animations for constructing ideas, relevant and well-labelled graphics, and links to other learning events in purple boxes. This has been implemented for year 1 and 2 medical students.

Inspired by the work of Mayer, ( 9 ) the template (Figure 2) aimed to reduce cognitive overload, which can otherwise compromise learning. The first step was minimising extraneous information (described by Mayer as ‘weeding’). Thus, the template reminds lecturers to include only relevant and well-labelled figures, and to keep the text concise within discrete boxes (Figure 2). Secondly, the template encourages strict colour-coding of boxes, providing the students with visual cues – (for example, green boxes were reserved for questions) – this ‘signalling’ can also reduce the cognitive load (Figure 2). Thirdly, maintaining temporal contiguity (well-timed appearance of new information), and spatial contiguity (consistency in, or good selection of, the location of information throughout a multimedia presentation) can also help reduce the cognitive load. Hence, the template suggests that animations and *PowerPoint* shapes should be used to ‘construct’ ideas (in conjunction with narration). ‘Aligning’ information correctly is also vital, as indicated by the deliberate positioning of text labels on the central diagram in the template (Figure 2). As the template evolves, however, it is crucial that the ‘individualised’ aspect of effective presentations be preserved; effective slides should contain formats suitable for students with varying learning styles ( 9 ).

Undoubtedly, the delivery of a lecture also influences the student engagement and recall ( 9 ); thoughtful narration can improve temporal contiguity, eliminate redundancy, allow ‘off-loading’ of information from visual to auditory channels, and ‘segment’ information. Body language, tone, and ‘signposting’ key points are all crucial. We, therefore, submitted a formal proposal for a ‘train the trainer’ course covering slide-building and lecture delivery. 

### Task 3 – Improvement and development of small-group teaching sessions

The students felt laboratory sessions could be more engaging; a framework for maximising the attractiveness and value of each session was developed. It had three aims – teaching scientific principles, research skills, and/or clinical medicine in context. All laboratory sessions in the BRS and Principles of Medicine (PoM) modules were evaluated within this framework. Indeed, setting clear objectives, running a focussed session, and ensuring each session meets the students’ needs, all improve small-group teaching sessions in medical schools ( 10 ).

We also designed our own small-group teaching sessions – a ‘Cells of the nervous system’ tutorial and a ‘Key histo-cytological features of the airways and heart’ laboratory session, both delivered using the e-learning platform *Lt kuraCloud*. We selected a topic, scoped the relevant TILOs, and identified a key learning point suitable for a small-group session. We proposed initial designs to the faculty, who, after checking feasibility and accuracy, approved our sessions. We authored these on *Lt kuraCloud*. For the ‘heart and lung’ session, we also designed an instructor-facing slide-show presentation. The ‘Cells of the nervous system’ session has already been implemented for first year students and the ‘Key histo-cytological features of the airways and heart’ session is set to be released following ‘beta-testing’ by students and staff.

## Discussion

### Strengths

This project was a success overall; all the three tasks were completed, and the team made significant progress towards the review. Faculty feedback revealed that students’ contributions were highly valuable. Since the project was open to all students and remuneration was offered, there was equal access and equitable recruitment. Indeed, the team was diverse with regards to ethnicity and gender, the importance of which is highlighted by Mercer-Mapstone and Bovill ( 11 ). The presence of a faculty member further diversified the team, and contributed to quality control. Since the students were in the first half of the MBBS course, their feedback was based on recent experience. The students also had prior roles in academic representation; they were accustomed to providing feedback. Crucially, the students were involved as ‘agents of change’ ( 12 ), not simply passive observers providing feedback. Indeed, the project allowed us to experience the session design for the first time; we now have a deeper understanding of the process and its challenges. The faculty and ICSM took important steps to lessen the imbalance of power that is often inherent to student-staff collaboration ( 13 ). This included verbal encouragement, allowing independence over our work, and emphasising that every suggestion is valuable. This was essential, especially given that faculty also had roles as assessors, which may, otherwise, have made us apprehensive about sharing our opinions. Review processes were grounded within student experiences and/or in the wider literature; this enabled evidence- and experience-based review. As students, we felt the project was personally developmental – we gained an understanding of educational theory. We also feel more comfortable in respectfully challenging the opinions of senior colleagues. Finally, we reflected throughout the project via contemporaneous discussions with faculty. 

### Limitations & further reflections

This collaboration required maximising productivity within a limited timeframe of just two weeks. Given more time, TILO reorganisation could have been improved by consulting the wider literature earlier and more extensively - for example using Skrbic’s ( 14 ) ‘SMART’ model as a core point of reference rather than an accessory tool. We could have also critiqued the submitted TILOs against Bloom’s taxonomy ( 7 ) more thoroughly, to fit the intended distribution more precisely. Furthermore, there was still greater scope for uniformity within the final list of TILOs; for example, some TILOs used the terms “causes and effects” whereas others used “pathophysiology”. During the design of the slides for large-group sessions, we could have taken accessibility and learning needs into account at an earlier stage and used a formal ‘checklist’, especially for pre-recorded video material ( 15 ). The design of small-group sessions may have been more efficient if we worked more ‘real-time’ with the faculty, rather than for feedback post-design, and if we had addressed image licensing earlier. Given more time, we would have taken a more active role in bringing our tasks to completion, for example overseeing TILO upload onto *Sofia*, or implementing the new teaching sessions ourselves. Finally, on a more general note, the project often required trial-and-error, especially with more creative tasks such as restructuring learning objectives. This is often an inherent challenge to student-staff collaboration, likely because the problems are unique and require novel approaches.

## Conclusions

Student-staff collaboration has significant productivity benefits. For example, we redesigned learning objectives, discussed slide design and refined/created teaching sessions.

Student-staff collaboration is developmental for the students involved, for example improving their understanding of educational theory.

Managing power dynamics is vital to optimise these collaborations.

Student availability, time constraints, and some ‘trial & error’ are potential challenges.

Overall, academic institutions are strongly recommended to organise and promote these collaborations in their own contexts.

## Acknowledgement

We would like to sincerely thank Dr James Moss (National Heart and Lung Institute) for his support & mentorship, and for creating Figures 1 and 2.

## Authors' contribution

Sh.J.K, L.N contributed to the conception and design of the work; the acquisition, analysis, or interpretation of data for the work. All Authors contributed in drafting and revising the manuscript critically for important intellectual content. All authors have read and approved the final manuscript and agree to be accountable for all aspects of the work in ensuring that questions related to the accuracy or integrity of any part of the work are appropriately investigated and resolved.

## Conflict of Interest:

None declared.
